# Identifying conditions for inducible protein production in *E. coli*: combining a fed-batch and multiple induction approach

**DOI:** 10.1186/1475-2859-5-27

**Published:** 2006-08-15

**Authors:** Marc G Aucoin, Virginie McMurray-Beaulieu, Frédéric Poulin, Eric B Boivin, Jingkui Chen, Francisc M Ardelean, Mathieu Cloutier, Young J Choi, Carlos B Miguez, Mario Jolicoeur

**Affiliations:** 1 Canada Research Chair on the Development of Metabolic Engineering Tools, Bio-P^2 ^Research Unit, Department of Chemical Engineering, Ecole Polytechnique de Montréal, P.O. Box 6079, Centre-Ville Station Montreal, Quebec, Canada; 2 Biotechnology Research Institute, National Research Council of Canada, 6100 Royalmount, Montreal, Quebec, Canada

## Abstract

**Background:**

In the interest of generating large amounts of recombinant protein, inducible systems have been studied to maximize both the growth of the culture and the production of foreign proteins. Even though thermo-inducible systems were developed in the late 1970's, the number of studies that focus on strategies for the implementation at bioreactor scale is limited. In this work, the bacteriophage lambda P_L _promoter is once again investigated as an inducible element but for the production of green fluorescent protein (GFP). Culture temperature, induction point, induction duration and number of inductions were considered as factors to maximize GFP production in a 20-L bioreactor.

**Results:**

It was found that cultures carried out at 37°C resulted in a growth-associated production of GFP without the need of an induction at 42°C. Specific production was similar to what was achieved when separating the growth and production phases. Shake flask cultures were used to screen for desirable operating conditions. It was found that multiple inductions increased the production of GFP. Induction decreased the growth rate and substrate yield coefficients; therefore, two time domains (before and after induction) having different kinetic parameters were created to fit a model to the data collected.

**Conclusion:**

Based on two batch runs and the simulation of culture dynamics, a pre-defined feeding and induction strategy was developed to increase the volumetric yield of a temperature regulated expression system and was successfully implemented in a 20-L bioreactor. An overall cell density of 5.95 g DW l^-1 ^was achieved without detriment to the cell specific production of GFP; however, the production of GFP was underestimated in the simulations due to a significant contribution of non-growth associated product formation under limiting nutrient conditions.

## Background

From a bioengineering standpoint, the integration of biological strain and process development is crucial to maximizing output. Optimisation of the production of recombinant protein in cells involves maximizing the number of cells that can massively produce the protein of interest properly. However, strong promoters constitute a metabolic burden on the host cell [1], and often the production of protein is at the expense of cell growth. Therefore strategies to separate the growth and production phases of a culture are required. Moreover, in the case of recombinant protein production, synthesizing recombinant proteins at the beginning of the culture may not be advantageous because many proteins are sensitive to proteases and, consequently, will be more susceptible to degradation before the final harvest [2]. One solution to this problem is to use an inducible promoter. Using small molecules or stresses that can modify the activation of the promoter, the start of protein production can be distinctively separated from the growth phase [3]. This also helps if the protein is toxic or detrimental to the growth of the host cell. Promoters used for high level-expression have been reviewed elsewhere [4]. Among the molecules used, isopropyl-β-D-thiogalactopyranoside (IPTG) is the most widely used in research. However, its use in large-scale production is scarce because of its high cost and its toxicity towards humans [5]. Lactose is also used as an inducer, as an alternative to IPTG. Lactose can activate the same promoters as IPTG while serving as a source of carbon for the bacteria [3].

An alternative to both IPTG and lactose is the use of a system with a temperature sensitive promoter/repressor. The most commonly used thermally induced promoters are the P_L _and P_R _promoters from the lambda phage. Below a temperature of 42°C, association with the cI repressor suppresses the activity of the promoter. At 42°C or above 37°C, the repressor is inactivated and the promoter recovers its activity.

When the inducible promoter is placed in front of the gene coding for the protein of interest, temperature will control the synthesis of this protein. Cells exposed to a temperature of 42°C will often exhibit diminished growth associated with the over-expression of foreign proteins [6] and should be considered when determining the induction point. Derepression of the phage lambda P_L _promoter has also been shown to increase the rate at which plasmid free segregants occur in the population [7]; therefore, prolonged periods under conditions that derepress the promoter can favour the propagation of plasmid free cells. A review of work conducted in the 1980's has indicated varied induction profiles that include 15 min temperature shifts from 30°C to 42°C returning to 37°C, a single shift to 42°C, as well as shifts that are product specific in duration [8]. For bioreactor runs a typical duration for induction conditions is of one hour; however, reports of shake flask studies have shown temperature shocks lasting as little as 2 minutes could yield optimal recombinant protein production when temperatures were lowered to 35°C for the remainder of the culture [8]. Two-minute temperature shocks may be unrealistic for the production at large scale where instantaneous temperature shifts are difficult to achieve. At large scale it is much more feasible to look at ramping the temperature up, allowing the temperature to stay at the induction temperature before bringing it back down to a secondary temperature. Step changes in temperature during continuous cultures, whereby cells are allowed to reach a steady state at 40°C before being brought up to 42°C, has been shown to yield high levels of recombinant protein activity [9]. Another approach to the study of thermo-inducible promoters has used a two-stage continuous process whereby cells grow in a first bioreactor kept at a temperature that represses the promoter, before flowing into a second bioreactor held at a higher temperature where production of recombinant protein ensues [10,11]. This has been shown to improve plasmid stability.

Recently the duration of conditions used for induction has been explored again [12]. From these experiments, it was concluded that a 20-minute exposure with a shift in temperature from 30°C to 39°C back to 37°C was optimal for the production of proteins. Furthermore, it has been reported that nutrient supply should also be considered when inducing the culture with temperature. Whitney et al. [13] showed that to sustain growth when using a temperature shift induction from 30°C to 42°C with *E. coli *NM989, a feed needs to be started to supplement the medium. However, it could be argued that growth may be at the expense of protein production, and limiting nutritional requirements for cell division may favour the production of the desired protein. Furthermore, lowering the temperature following the initial temperature shock should slow down the cellular metabolism thus lowering the nutritional requirements.

In this work, the characterization of temperature inducible processes was studied using green fluorescent protein (GFP). GFP was chosen as the target protein, mainly due to its ease of quantitation. Temperature cycling between regimes known to promote and inhibit protein synthesis was examined in addition to the induction point, the duration of induction and the number of inductions. Multiple exposures to the "inducer" has not been reported in the literature, to our knowledge, with the exception of a triple dosage of lactose, which was used as inducing agent [3]. In those experiments, the addition of lactose in three consecutive pulses was used to avoid toxicity and to constantly keep an inducing concentration of lactose in the medium. For this reason, it is of interest to see whether any positive effect can be achieved by allowing the system to return to a state known to inhibit the production of foreign protein and to see whether simple models can reflect this mode of operation. Temperature cycling has been suggested to improve plasmid stability in batch cultures even though it has provided sub-optimal results for continuous two-stage cultures [10]. Also in this work, the culture of *E. coli *at 37°C, which has often been used as a final cycle temperature, is investigated as mid-range temperature. The objective of this work was to maximize the volumetric production of GFP in *E. coli *pND-GFP.

## Model development

For this work, a Monod kinetic model based on glucose as growth-limiting substrate was used. The model used combines terms for cell growth, glucose consumption, product formation and volume variation with two sets of parameters related to the pre-induction and post-induction periods.

The cell growth is represented by:

dXdt=μX−(FV)X     (1)
 MathType@MTEF@5@5@+=feaafiart1ev1aaatCvAUfKttLearuWrP9MDH5MBPbIqV92AaeXatLxBI9gBaebbnrfifHhDYfgasaacH8akY=wiFfYdH8Gipec8Eeeu0xXdbba9frFj0=OqFfea0dXdd9vqai=hGuQ8kuc9pgc9s8qqaq=dirpe0xb9q8qiLsFr0=vr0=vr0dc8meaabaqaciaacaGaaeqabaqabeGadaaakeaadaWcaaqaaiabdsgaKjabdIfaybqaaiabdsgaKjabdsha0baacqGH9aqpiiGacqWF8oqBcqWGybawcqGHsisldaqadiqaamaalaaabaGaemOrayeabaGaemOvayfaaaGaayjkaiaawMcaaiabdIfayjaaxMaacaWLjaWaaeWaceaacqaIXaqmaiaawIcacaGLPaaaaaa@3FCE@

μ=μmax⁡SKS+S     (2)
 MathType@MTEF@5@5@+=feaafiart1ev1aaatCvAUfKttLearuWrP9MDH5MBPbIqV92AaeXatLxBI9gBaebbnrfifHhDYfgasaacH8akY=wiFfYdH8Gipec8Eeeu0xXdbba9frFj0=OqFfea0dXdd9vqai=hGuQ8kuc9pgc9s8qqaq=dirpe0xb9q8qiLsFr0=vr0=vr0dc8meaabaqaciaacaGaaeqabaqabeGadaaakeaaiiGacqWF8oqBcqGH9aqpdaWcaaqaaiab=X7aTnaaBaaaleaacyGGTbqBcqGGHbqycqGG4baEaeqaaOGaem4uamfabaGaem4saS0aaSbaaSqaaiabdofatbqabaGccqGHRaWkcqWGtbWuaaGaaCzcaiaaxMaadaqadiqaaiabikdaYaGaayjkaiaawMcaaaaa@3F11@

S refers to the extra-cellular glucose concentration, which is assumed to be the growth-limiting substrate. The increase in the working volume is followed using:

dVdt=F     (3)
 MathType@MTEF@5@5@+=feaafiart1ev1aaatCvAUfKttLearuWrP9MDH5MBPbIqV92AaeXatLxBI9gBaebbnrfifHhDYfgasaacH8akY=wiFfYdH8Gipec8Eeeu0xXdbba9frFj0=OqFfea0dXdd9vqai=hGuQ8kuc9pgc9s8qqaq=dirpe0xb9q8qiLsFr0=vr0=vr0dc8meaabaqaciaacaGaaeqabaqabeGadaaakeaadaWcaaqaaiabdsgaKjabdAfawbqaaiabdsgaKjabdsha0baacqGH9aqpcqWGgbGrcaWLjaGaaCzcamaabmGabaGaeG4mamdacaGLOaGaayzkaaaaaa@37E2@

dSdt=(FV)(Sf−S)−μXYX/S−(YP/Xμ+β)XYP/S−msX     (4)
 MathType@MTEF@5@5@+=feaafiart1ev1aaatCvAUfKttLearuWrP9MDH5MBPbIqV92AaeXatLxBI9gBaebbnrfifHhDYfgasaacH8akY=wiFfYdH8Gipec8Eeeu0xXdbba9frFj0=OqFfea0dXdd9vqai=hGuQ8kuc9pgc9s8qqaq=dirpe0xb9q8qiLsFr0=vr0=vr0dc8meaabaqaciaacaGaaeqabaqabeGadaaakeaadaWcaaqaaiabdsgaKjabdofatbqaaiabdsgaKjabdsha0baacqGH9aqpdaqadiqaamaalaaabaGaemOrayeabaGaemOvayfaaaGaayjkaiaawMcaamaabmGabaGaem4uam1aaSbaaSqaaiabdAgaMbqabaGccqGHsislcqWGtbWuaiaawIcacaGLPaaacqGHsisldaWcaaqaaGGaciab=X7aTjabdIfaybqaaiabdMfaznaaBaaaleaacqWGybawcqGGVaWlcqWGtbWuaeqaaaaakiabgkHiTmaalaaabaWaaeWaceaacqWGzbqwdaWgaaWcbaGaemiuaaLaei4la8IaemiwaGfabeaakiab=X7aTjabgUcaRiab=j7aIbGaayjkaiaawMcaaiabdIfaybqaaiabdMfaznaaBaaaleaacqWGqbaucqGGVaWlcqWGtbWuaeqaaaaakiabgkHiTiabd2gaTnaaBaaaleaacqWGZbWCaeqaaOGaemiwaGLaaCzcaiaaxMaadaqadiqaaiabisda0aGaayjkaiaawMcaaaaa@6047@

The first term represents the increase in the glucose concentration due to the feeding. F is set to 0 for a culture operated in a batch mode. The second, third and final terms correspond to the glucose used to support growth, to produce GFP and to maintain cells, respectively.

Finally, it was hypothesized that GFP production would follow a mixed-growth-associated product formation kinetic:

dPdt=(YP/Xμ+β)X−FVP     (5)
 MathType@MTEF@5@5@+=feaafiart1ev1aaatCvAUfKttLearuWrP9MDH5MBPbIqV92AaeXatLxBI9gBaebbnrfifHhDYfgasaacH8akY=wiFfYdH8Gipec8Eeeu0xXdbba9frFj0=OqFfea0dXdd9vqai=hGuQ8kuc9pgc9s8qqaq=dirpe0xb9q8qiLsFr0=vr0=vr0dc8meaabaqaciaacaGaaeqabaqabeGadaaakeaadaWcaaqaaiabdsgaKjabdcfaqbqaaiabdsgaKjabdsha0baacqGH9aqpdaqadiqaaiabdMfaznaaBaaaleaacqWGqbaucqGGVaWlcqWGybawaeqaaGGacOGae8hVd0Maey4kaSIae8NSdigacaGLOaGaayzkaaGaemiwaGLaeyOeI0YaaSaaaeaacqWGgbGraeaacqWGwbGvaaGaemiuaaLaaCzcaiaaxMaadaqadiqaaiabiwda1aGaayjkaiaawMcaaaaa@46ED@

The first term (*Y*_*P*/*X*_*μX*) corresponds to the growth-associated product while the second term (β *X*) represents non-growth-associated product formation. The last term considers the dilution caused by feeding. This assumption is broad and can be used to identify if in fact the non-growth associated term is negligible as suggested by others [8].

## Results

### Influence of temperature on growth and promoter induction

**Figure 1:**
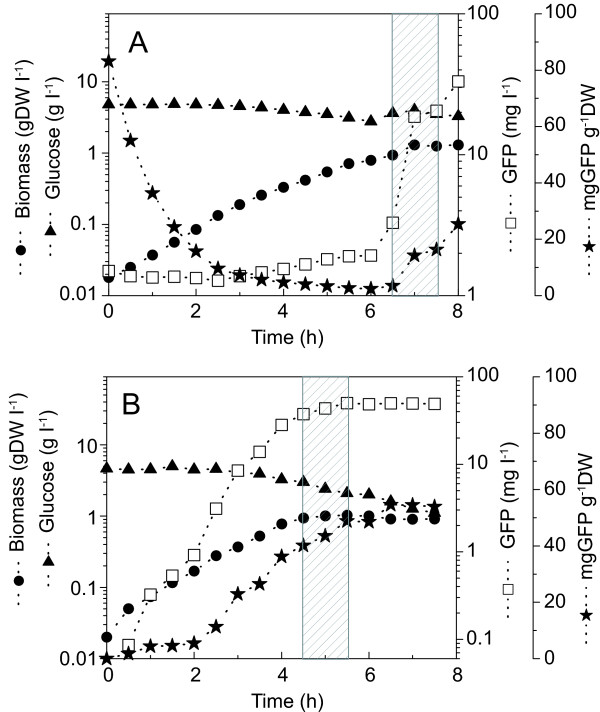
Promoter control in 20L cultures done at 30°C and 37°C. A. Batch culture at 30°C till 6 hours. At 6 hours, a feed was started as described in the text. Induction to 42°C started at 6.5 hours and lasted one hour, as indicated by the gray box. B. Batch culture at 37°C. Induction to 42°C started at 4.5 hours and lasted for one hour, as indicated by the gray box.

Two cultures were performed in a 20-L bioreactor at temperatures of 30°C (Fig. 1A) and 37°C (Fig. 1B) to study promoter regulation with temperature. The exponential growth phase started at inoculation for both cultures with a similar value of the specific growth rate of 0.77 ± 0.03 d^-1^. It should be noted that glucose was not limiting in either culture with a residual concentration at the end of the cultures of 2.7g l^-1 ^and 1.1 g l^-1 ^at 30°C and 37°C respectively. During the exponential growth phase, the apparent biomass yield coefficient was slightly higher at 37°C (Y_*X*/*SApp *_= 0.40 ± 0.05) than that at 30°C (Y_*X*/*SApp *_= 0.35 ± 0.01). This was calculated between t = 1.5 and t = 4 hours for the culture done at 37°C and t = 1.5 hours to t = 6 hours for the culture done at 30°C, to avoid error introduced by the minute changes in glucose at very low cell densities and to capture as much of the exponential phase prior to induction or feeding. The growth rate of the culture carried out at 37°C seemed to have attenuated after the first half hour of culture. Although it is possible that this change in growth rate may be an artefact introduced by measurement error, it was concomitant to a rapid increase in GFP signal. Furthermore, when the cells were grown at 37°C, GFP production started without the need for increasing the temperature to 42°C and a cell content of 40 mg GFP g^-1 ^DW was already reached before the temperature induction. The promoter for GFP production leaked significantly at 37°C. Prior to inducing the synthesis of protein in the culture grown at 30°C by raising the temperature to 42°C, a feed consisting of 10 × medium concentrate was started to see if additional nutrients at the time of induction could alleviate the strain of the elevated temperature. This feed did not affect the pre-induction culture conditions or the regulation of the promoter at 30°C. A small increase in biomass was observed at the time of the addition. Raising the temperature of the culture to 42°C for a period of 1 hour, to induce GFP production, coincided in a reduced growth rate for the culture performed at 30°C (μ = 0.025 ± 0.02 d^-1^) and growth cessation for the culture at 37°C. It was not clear through these experiments the effect of the feed on events post-induction. In the case of the 30°C culture, GFP levels prior to induction were confounded with the background signal from the medium suggesting that the promoter was not leaking at 30°C. However, after induction, the level of GFP increased abruptly reaching 38 mgGFP g^-1 ^DW 2 h post-induction. There was no noticeable effect of a temperature induction at 42°C on the specific cell GFP content for the 37°C culture; the trend for GFP production, leading to a maximum cell content of 54 mgGFP g^-1 ^DW, did not seem to be due to the induction.

### Effect of successive inductions on promoter efficiency

**Figure 2:**
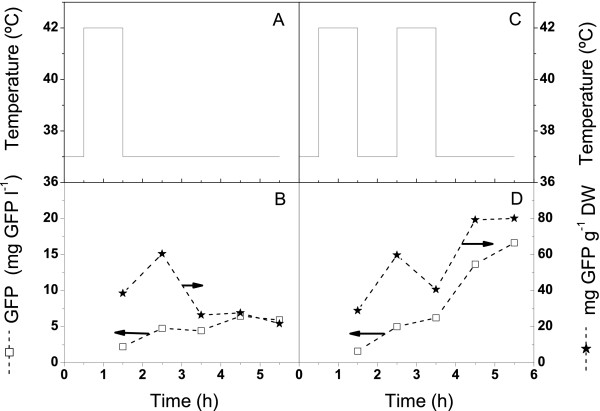
Investigation on the effect of multiple temperature inductions at 42°C on GFP production. Cultures were performed at 37°C in a shake flasks as described in Materials and Methods. Samples were induced at 42°C for a period of 1 hour as described and then returned to 37°C.

The behaviour of the promoter at 37°C presented in Figure 1B was intriguing and experiments were thus conducted to study whether the time of promoter induction at 42°C could be useful. Shake flask cultures were performed to evaluate an optimal induction time and temperature profile for GFP expression. For the shake flask cultures at 37°C, only the flasks started at 0.5 hour post inoculation (i.e. samples taken from the bioreactor (Fig. 1B) at 0.5 hour for the induction study), significantly showed the effect of the temperature shift from 37°C to 42°C (Fig. 2), and resulted in an increase in GFP concentration. All other cultures exposed to 37°C for longer periods of time and exposure thereafter to a temperature of 42°C did not result in an increase in GFP production that could be associated with an exposure to the induction temperature (data not shown). Figure 2 also indicates that a dual induction strategy or cycling between temperatures may be beneficial for the production of GFP. A dual induction profile resulted in a production nearly three times greater than that achieved with a single induction.

**Figure 3:**
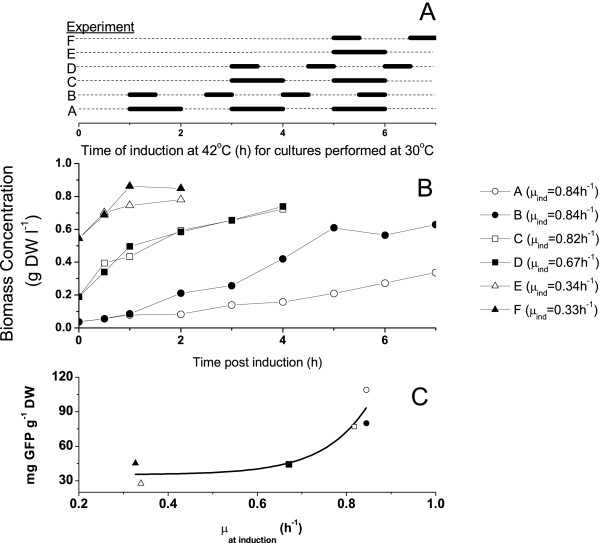
Investigation of the effect of multiple temperature inductions at 42°C on GFP production. Cultures were performed at 30°C in shake flasks as described in Materials and Methods. Samples were taken from shake flasks, induced as described and then cultured at 30°C. A. Induction sequence for the six experiments. B. Biomass production after induction. C. Relationship between specific productivity and pre-induction growth rate.

Since operating under "leaky" promoter conditions is not desired, the effect of successive inductions from a temperature that represses the gene was also investigated. Single and multiple temperature shifts between 30°C to 42°C (Fig 3A) were applied to the set of sub-cultures removed from bioreactor operated at 30°C (Fig. 1A). In this study, no significant change in the production of GFP could be observed beyond two inductions for all cultures described in Figure 3. Biomass concentrations continued to increase after induction at a rate that depended on the growth rate when the initial induction was applied. Figure 3B shows the post-induction dry cell mass concentrations obtained in the shake flask cultures at 30°C for various induction profiles such as defined in Figure 3A. Although there was some evidence that a 1-hour exposure to a temperature of 42°C resulted in a decrease in biomass synthesis, higher specific production were achieved with the longer exposure time (Fig. 3C). Finally, results also suggested that the cell specific production of GFP may be a function of the cells growth rates prior to induction (Fig. 3C).

### Calibration of the model parameters

**Figure 4:**
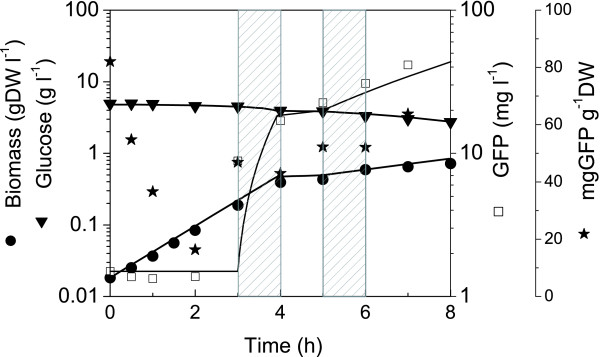
Data for model calibration. Cultures performed in shake flask and induced at 3 h and 5 h for 1 h from 30°C to 42°C, as indicated by the gray boxes. Data were used for the determination of the model kinetic parameters showed in Table 1. Symbols refer to experimental data and lines to model simulation (model fitting).

In an attempt to design a process scheme that would increase the volumetric yield of GFP, numerical simulations of the production of GFP in *E. coli *were carried out. The simulations considered the dynamics of biomass growth, glucose consumption, GFP production and volume change (eq.1 to 5). The simulations were carried out using the ode15s solver in Matlab v6.5.1 (MathWorks). The time domain was divided into two distinct phases for pre-induction and post-induction. A different set of kinetic parameters was employed for each phase (Table 1). Eight parameters were estimated including maximum growth rates before and after induction, biomass yields on substrate before and after induction, substrate affinity coefficient (based on Michaelis-Menten kinetics), maintenance factor and two constants for the semi-growth associated product formation (turned on at induction). The kinetic parameters, listed in Table 1, were obtained by minimizing the error between the numerical simulations and the values obtained for biomass, glucose and GFP concentrations in the shake flask cultures. The experimental data presented in Figure 4 were used to calibrate model parameters and then the model was used to control a fed-batch culture as discussed in the following sections. Although the culture presented in Figure 4 underwent a dual induction profile, for the purpose of model development, only the first induction was considered. This culture was chosen because it was induced in the mid-exponential phase of growth. The minimization algorithm used was the fminsearch function in Matlab v6.5.1 (MathWorks). Initial values for the parameters used in the simulations were found in the literature or estimated from the experimental data calculated from the cultures.

**Table 1:** Kinetic parameters determined using batch shake flask cultures showed in Figure 4

	**Before induction**	**After induction**	
*K*_*s*_	0.4	0.4^a^	(g glu l^-1^)
*m*_*s*_	0.0025	0.0025^a^	(g glu g DW^-1^h^-1^)
μ_max_	0.9	0.2	(h^-1^)
*Y*_*X*/*S*_	0.6	0.33	(g DW g^-1^glu)
*Y*_*P*/*S*_	0	50	(mg GFP g^-1^glu)
*Y*_*P*/*X*_	0	66.92	(mg GFP g^-1 ^DW)
β	N/A	0.10	(mg GFP g DW^-1 ^h^-1^)

^a ^*K*_*s *_and *m*_*s *_determined before induction were used

Parameters that differed between the two phases were the growth rate and the biomass yield coefficient. A drop in growth, post-induction, was seen in all cultures studied for this work. To obtain a proper simulation of the cultures at 30°C, a time delay was incorporated in the model at the time of induction. The delay was set to 1 hr and was based on the response observed from the shake flask cultures. Temperature induction, however, caused a time lag in cell division but not in GFP production.

### Fed-batch strategy for increased GFP production

A fed-batch strategy for the pre-induction phase was developed from model simulations by setting a constant glucose concentration at a low but non-limiting concentration. Based on the results from the shake flask studies, a glucose concentration of 3 g l^-1 ^was selected because a non-limiting glucose concentration showed to lead to a high pre-induction growth rate, and then to a high productivity post-induction. It was also decided to feed a concentrated medium that contained both glucose and nutrient broth to avoid limitation from other medium constituents. The simulated feed rate profile to maintain a glucose set-point concentration at 3gl^-1 ^was approximated by step changes for manual changes in pump settings. The culture was then induced (30°C to 42°C) twice following a different strategy after each induction. A first induction was performed at 5 h followed by non-limiting glucose conditions. Feed rate at "t" was then estimated from model simulation using experimental data at "t". The second induction, which was performed at 9 h, was followed by glucose limiting conditions. Each induction cycle consisted of ramping the temperature from 30°C to 42°C, then maintaining the temperature at 42°C for 1 hour before bringing the temperature back to 30°C.

### Performance of the fed-batch strategy

**Figure 5:**
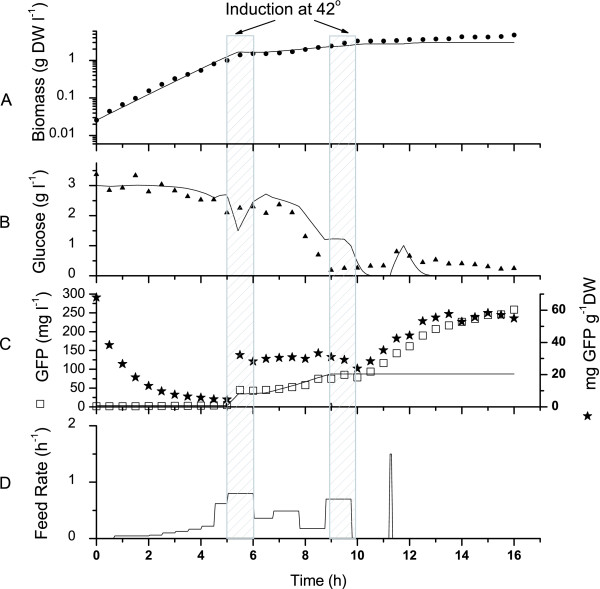
Fed-batch culture performed using the model to determine the feed rate. The culture was performed at 30°C in a 20-l bioreactor with control of dissolved oxygen. Inductions were performed at 5 h and 9 h for 30 min from 30°C to 42°C, as indicated by the gray boxes. Experimental data (points) and model simulation (continuous lines) of the "optimal" culture.

The feed rate profile estimated from model simulations maintained both a constant exponential growth rate (μ = 0.78 ± 0.001) and a glucose concentration set at ~3 g l^-1 ^(Fig. 5). At 4.5 h, however, the measured glucose concentration showed a slight tendency to decrease so the feed rate was increased for 30 min at a higher level than the estimated one. Inoculated cells showed to contain GFP but these values (up to ~300 mg g^-1^DW) may be overestimated when dividing measured GFP in the cell suspension by the low biomass concentration for the first two hours after inoculation (similar to the overestimation seen in Figure 1A).

The culture was first induced in the exponential phase (5 h). Then, the cells fell into a latency phase prior to recovering at 7 h with an attenuated growth rate (0.17 ± 0.02). The estimated feed rate then had difficulties maintaining the glucose concentration at 3 g l^-1 ^because when cell growth restarted the glucose consumption rate was higher than expected. The glucose concentration then dropped and became limiting at 8 h. Interestingly, the culture broth content in GFP followed biomass behaviour but the specific cell GFP content rapidly reached a plateau during the temperature induction and then stayed highly constant at 30 mg g^-1^DW from 5.5 h to 9 h. This specific cell GFP content was similar to that measured for shake flask cultures performed at 30°C and induced at 42°C (Figs. 1A and 3C) but lower than cultures performed at 37°C (also induced at 42°C) (Figs. 1B and 2). During the period following the first temperature induction (5 – 9 h), the model simulation predicted biomass concentration as well as GFP production. However, the model has under-estimated the required feed rate resulting in a higher predicted glucose concentration than observed experimentally.

**Figure 6:**
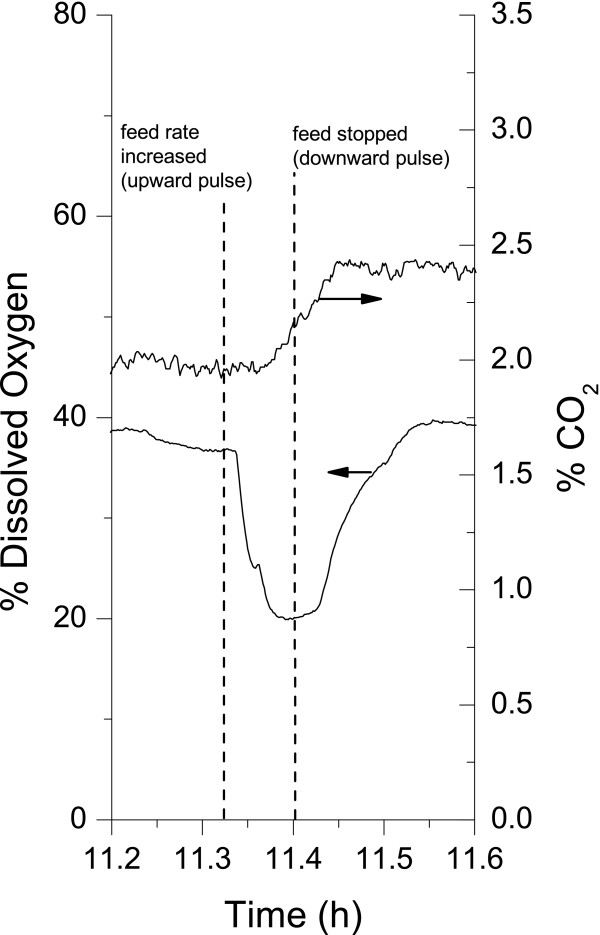
Assays for glucose limiting status of the 20-l bioreactor culture (Figure 5). On-line data corresponding to a feed pulses used to assess the state of the cells with respect to feeding: the effect of glucose feed pulse on dissolved oxygen and CO_2 _(agitation was controlled by computer).

The cells were then re-induced at 9 h but now under glucose limiting conditions. A decrease in the specific GFP production (mg GFP/g DW) was observed immediately after the induction (Fig. 5C). This was probably due to an increase in biomass as the temperature increased (Fig. 5A), which resulted in the dilution of the GFP protein units between mother and daughter cells. Despite the slight reduction during the induction period the specific GFP cell content started to increase (at 10 h) when the temperature was returned to 30°C. This increase continued until 13.5 h while the biomass reached a plateau and glucose stayed at limiting conditions. A maximum specific GFP cell content of 60 mg GFP g^-1 ^DW was reached, which was twice that measured after the first induction performed under non-limiting glucose conditions. The plateau observed for the cell specific GFP content at 14 h may be due to a combination of prolonged glucose limiting conditions and a decrease in the effect of the temperature induction. A feed pulse test, to verify for either feed limitation or acetate production as described by Akesson et al. [14], was done at t = 11.3 h and confirmed that glucose was limiting (Fig. 6). With the glucose being exhausted in the simulation, GFP production beyond the second induction was not predicted, thus showing that the non-growth associated product formation model was highly underestimated. The final biomass concentration attained was 5.95 g DW l^-1 ^and the concentration of GFP reached 273.14 mg l^-1^. With a pre-defined feeding strategy, the biomass was increased 4 fold compared to previous cultures. Overall GFP production was ~5.5 times greater than that achieved in the batch cultures. The apparent product yield based on the cell specific production, Y_*P*/*XApp*_, was also increased over previous bioreactor cultures. It was 20.5% higher than the fed-batch culture at 30°C and 17% higher than the batch culture at 37°C.

## Discussion

An alternative genetic strategy in the development of a temperature inducible process exists when the λ P_L _promoter is placed in front of a gene that sets the frequency of replication from the origin of replication. In that scenario increases in temperature lead to the runaway production of plasmid and the concomitant increase in foreign protein production [15-17]. However, the increases in plasmid production are detrimental to the cell and the highest protein yields are not achieved if the plasmid replication is not brought under control (temperature downshift). In fact, Larsen et al. (1984) reported a four times greater yield when the culture was brought back to 38°C after subjecting the culture to 42°C for 45 minutes (culture initially at 30°C) [15].

Not unlike the system above, raising the temperature to 42°C is detrimental to cell growth for the system examined within this article. The difference is that with control of the replicon, it can be foreseen that multiple cycles based on the doubling time may be beneficial in sustaining cell viability while maximizing production of foreign protein. Each doubling after the initial exposure would result in the dilution of the plasmid because each daughter cell would keep, on average, one half of the available plasmid copies. Furthermore, it is conceivable that such a strategy would also benefit from different "induction" periods, with shorter exposure periods resulting in similar specific plasmid content (plasmids/cell). For a system whereby only protein synthesis is affected, such a strategy is not as easily conceivable; however, may be beneficial in terms of plasmid stability [7].

Depending on the promoter sequence, regulation with respect to culture temperature may differ for different plasmid constructs. Culturing the cells at 37°C allowed the concurrent production of GFP but caused them to enter their stationary phase at a lower cell density than did culturing them at 30°C. It is also known that sustained elevated temperatures can result in plasmid segregation [7], which could promote the propagation of plasmid free cells. It was therefore questioned whether a strategy of returning to 37°C after the initial induction would be an appropriate strategy. Furthermore, it was questioned whether returning to 30°C would sustain production or whether a strategy of re-induction would be necessary and advantageous.

### Induction strategy: when and how many?

Although the rate of synthesis was higher and the substrate utilization seemed better at 37°C (Y_X/SApp, 37°C _= 0.40 ± 0.05 vs Y_X/SApp, 30°C _= 0.35 ± 0.01), a strategy based on the "leakiness" of a promoter was thought to be counter-intuitive for non-continuous cultures; therefore, starting the culture under conditions that were known to truly inhibit was desired. The "leakiness" of the promoter could, however, be beneficial to regulate recombinant expression at specific levels especially in continuous cultures as proposed by others [9]. In this work there was evidence that multiple cycles, whether it be between 37°C and 42°C or 30°C and 42°C resulted in increased product yield. The type of cycling described in this work is readily achievable in a bioreactor because the operation of a bioreactor is conducive to temperature ramping. The idea of using a cyclical pattern has been previously explored as a way to inactivate de novo synthesized repressor molecules in a continuous culture [10], however, the temperature profile never dropped below 39°C. In our group, we have seen that returning to 30°C yields better results than when the culture is maintained at a higher temperature (37°C) after exposure to a temperature of 42°C (data not shown).

In this work there was evidence that higher pre-induction growth rates resulted in higher specific productivities; however, the multitude of conditions examined do not lead to a definite conclusion. This result would be is consistent with Ryan et al. (1996), who also showed that cells growing at higher growth rates before they are subjected to high temperatures yield higher cloned gene expression [18]. Faster-growing cells are thought to be more resourceful in adapting to stressful conditions in terms of product synthesis and therefore will dictate the optimal time for inducing the culture. This point means that it may be necessary to sacrifice higher cell densities unless appropriate feeding or operating strategies, like fed-batch or chemostat operation, can be designed to maintain the culture at high growth rates. In another study, however, the growth rate before or during induction was shown to have no influence on production, while a greater biomass concentration gave higher recombinant protein activity [19]. Considering the goal of optimising volumetric yield of recombinant protein, increasing cell density and maintaining high pre-induction growth rates were desired.

### Increasing cell density and volumetric yield

Using a simple feeding strategy combined with the use of multiple inductions, starting in the exponential growth phase, the volumetric yield was significantly enhanced without compromising the specific yield on biomass. Interestingly we observed that even though growth was attenuated late in the culture, due likely to a mix of nutritional limitations and the synthesis of foreign proteins, the increase in GFP was quite significant. This was further shown in the apparent product on biomass yield coefficient (Y_*P*/*XApp*_), which nearly doubled between the second induction and the end of the culture. In fact, as seen in Figure 5, the specific cell content of GFP (mg GFP g^-1 ^DW) increases steadily after the second induction between t= 10 and t= 13.5 hours, during glucose limiting conditions. Although it has been shown that there is a high nutritional demand at high cell densities, we see here that even with glucose in limiting amounts, production of GFP was not hindered. Future work will investigate the levels of glucose and other nutrients that need to be maintained in the culture after induction to maximize recombinant protein production. Furthermore, it is of interest to establish the cause of the plateau that occurs beyond t= 13.5 hours.

## Conclusion

In this study it has been shown that simultaneous growth and GFP production can be achieved when cultivating *E. coli *pDN-GFP at 37°C. Furthermore, continuous high-level expression was not achieved with cycling once from 30°C to 42°C and back to 30°C. Multiple inductions enhanced the production of the temperature inducible product. A refined strategy of re-induction based on the doubling time of the bacteria post-induction is an avenue of great interest. Simple modelling techniques can be used to describe the system and be used to formulate a feeding strategy that increases the volumetric yield. Allowing for time domains within the process allows more flexibility in modelling the culture dynamics. Future work will also investigate if product formation models need to be linked to nutrient levels i.e. separate models for non-limiting and limiting conditions. A sensitivity analysis will then be performed to identify the significant model parameters.

## Methods

### Microorganism and medium

**Figure 7:**
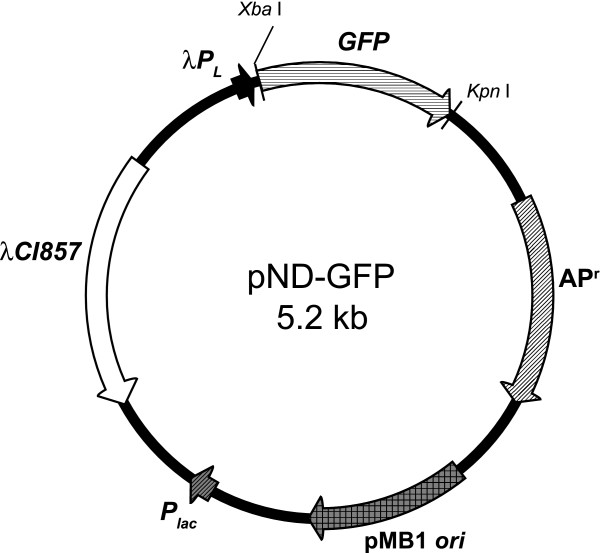
Plasmid map of *E. coli *pDN-GFP.

Manipulations and sequencing of DNA were carried out using standard methods [20]. The 0.8 kb DNA fragment carrying the gfp gene was amplified from pCM110-GFP [21] using primers GFP-F-*Xba*I (5'-GTCTAGAATGGCTAGCAAAGGAGAAGAAC-3') and GFP-R-*Kpn*I (5'-CGGTACCTCAGTTGTACAGTTCATCCATGC-3'). The restriction sites have been underlined. The PCR product was then cloned into pCR2.1-TOPO vector generating pCR2.1-GFP. The *Xba*I-*Kpn*I fragment of GFP gene was then cloned into the thermo-inducible vector pND-GFP under the control of the promoter, λ *P*_*L*, _to form pND-GFP (Figure 7). *E. coli *strain TOP10 harboured the temperature-inducible plasmid, pND-GFP was used for all cell cultures.

Medium containing 8 g l^-1 ^nutrient broth (BD Bioscience, Maryland, USA), 5 g l^-1 ^sodium chloride, and glucose was used.

### Pre-cultures

The plasmid bearing cells were plated onto agar medium containing ampicilin. Colonies were collected and inoculated into shake flasks with a 100 ml working volume and were incubated overnight at 25°C with an orbital agitation rate between 220 and 250 rpm. The medium previously described was used.

### Batch and fed-batch cultures

One batch and two fed-batch experiments were carried out in a 20-l bioreactor (Bioengineering, Switzerland). For the batch culture, 300 ml of pre-culture containing approximately 5.5 g DW l^-1 ^of biomass were used to inoculate the bioreactor filled with 12 l of medium and 1 ml of Mazu DF 204 antifoam (BASF, New Jersey, USA). The initial glucose concentration was 5 g l^-1^. The method followed for the fed-batch cultures is the same as previously described for the batch mode except that 500 ml of inoculum was used instead of 300 ml. For the first fed-batch culture (Fig. 1A), the initial concentration of glucose in the medium was 5 g l^-1^. One litre of a concentrated feed (10 ×) containing 80 g l^-1 ^nutrient broth, 5 g l^-1 ^sodium chloride and 50 g l^-1 ^glucose was added over a period of one hour after the culture had reached 6 hours. For the second fed-batch culture (Fig. 5), the initial bioreactor volume was 10 l and the initial glucose concentration in the medium was lowered to 3 g l^-1^. Four litres of a concentrated feed containing 80 g l^-1 ^nutrient broth, 5 g l^-1 ^sodium chloride and 30 g l^-1 ^glucose were added according to a predetermined feed strategy. The duration of the culture was 17 hours. It should be noted that a limitation in the concentration of the feed solution is the solubility of the nutrient broth. We report that there were no solubility issues associated with making a medium solution containing 80 g l^-1 ^nutrient broth.

For cultures carried out in bioreactors, pH and dissolved oxygen were controlled at 7 and 45%, respectively. Dissolved oxygen was initially high and decreased to the controlled value within 1.5 hours after inoculation.

### Shake flask cultures

To study the induction of the cells, 50 ml samples were taken from a bioreactor at various times and grown in shake flasks. The shake flask cultures were incubated at the same temperature as the initial bioreactor from which they were taken. The shake flask cultures were induced up to four times for periods of either 30 or 60 min at 42°C in a separate incubator. Samples from these cultures were taken every hour and were analysed for glucose, biomass and GFP according to the analytical methods described below. The biomass concentration in the shake flask cultures was determined by optical density only.

### Analysis

Samples from the bioreactor were taken every thirty minutes for glucose, biomass and GFP analysis. The glucose concentrations remaining in the medium were established by an enzymatic test using Infinity Glucose Reagent (Sigma, ref. [17, 18, 19, 20, 21, 22, 23, 24, 25]). The biomass concentrations were determined by optical density and dry weight measures. The GFP concentrations were measured using a SpectraFluor Plus fluorescence spectrophotometer (Tecan, North Carolina, U.S.A.).

## Abbreviations

DW Dry weight

*F *Feed rate (h^-1^)

Glu Glucose

*K*_*s *_Glucose affinity constant (g glu l^-1^)

*m*_*s *_Maintenance coefficient (g glu g^-1 ^DW h^-1^)

*V *Liquid volume in the reactor (l)

*P *Product (GFP) concentration (mg l^-1^)

*S *Extracellular glucose concentration (g glu l^-1^)

*S*_*f *_Glucose concentration in feed (g glu l^-1^)

*t *Time (h)

*X *Biomass concentration (g DW l^-1^)

*Y*_*P*/*S *_Product yield on substrate coefficient (mg GFP g^-1^glu)

*Y*_*P*/*X *_Product yield on biomass coefficient (mg GFP g^-1 ^DW)

*Y*_*X*/*S *_Biomass yield on substrate coefficient (g DW g^-1^glu)

β Non-growth associated product formation constant (mg GFP g DW^-1 ^h^-1^)

μ Specific growth rate (h^-1^)

μ_*max *_Maximum specific growth rate (h^-1^)

## Competing interests

The author(s) declare that they have no competing interest.

## Authors' contributions

MGA, VMB, FP, EBB, JC, FMA carried out the experiments and participated in drafting a document describing the work at hand. MC assisted in the running of the experiments and did the analysis of the green fluorescent protein. YJC and CBM constructed the plasmid and transformed cells used in this study. MGA and MJ have designed the experiments presented in this manuscript. This work has been performed as part of a graduate course on Cell Culture Modeling (GCH6302) coordinated by Pr. M. Jolicoeur, in the Department of  Chemical Engineering at École Polytechnique de Montréal.

## References

[B1] Reischer H, Schotola I, Striedner G, Pötschacher F, Bayer K (2004). Evaluation of the GFP signal and its aptitude for novel on-line monitoring strategies of recombinant fermentation processes. Journal of Biotechnology.

[B2] Schmidt M, Babu KR, Khanna N, Marten S, Rinas U (1999). Temperature-induced production of recombinant human insulin in high cell-density cultures of recombinant Escherichia coli. Journal of Biotechnology.

[B3] Gombert AK, Kilikian BV (1998). Recombinant gene expression in Escherichia coli cultivation using lactose as inducer. Journal of biotechnology.

[B4] Hanning G, Makrides SC (1998). Strategies for optimizing heterologous protein expression in Escherichia coli. Trends in Biotechnology.

[B5] Menzella HG, Ceccarelli EA, Gramajo HC (2003). Novel Escherichia coli strain allows effiecient recombinant protein production using lactose as inducer. Biotechnology and Bioengineering.

[B6] Lim HK, K-H J (1998). Improvement of heterologous Protein Productivity by Controlling Postinduction Specific Growth Rate in Recombinant Escherichia coli under control of the PL Promoter.. Biotechnology Progress.

[B7] Siegel R, Ryu DDY (1985). Kinetic study of instability of recombinant plasmid pPLc23trpAl in E. coli using two-stage continuous culture system. Biotechnology and Bioengineering.

[B8] Gupta JC, Jaisani M, Pandey G, Mukherjee KJ (1999). Enhancing recombinant protein yields in Escherichia coli using the T7 system under the control of heat inducible lPL promoter. Journal of Biotechnology.

[B9] Villaverde A, Benito A, Viaplana E, Cubarsi R (1993). Fine regulation of cI857-Controlled Gene Expression in Continuous Culture of Recombinant Escherichia coli by Temperature. Applied and Environmental Microbiology.

[B10] Hortacsu A, Ryu DDY (1990). Optimal temperature control policy for a two-stage recombinant fermentation process. Biotechnology Progress.

[B11] Lee SB, Ryu DDY, Seigel R, Park SH (1988). Performance of recombinant fermentation and evaluation of gene expression efficiency for gene product in two-stage continuous culture system. Biotechnology and Bioengineering.

[B12] Chao YP, Law WS, Chen PT, Hung WB (2002). High production of heterologous proteins in Escherichia coli using the thermo-regulated T7 expression system. Applied Microbiology and Biotechnology.

[B13] Whitney GK, Glick BR, Robinson CW (1989). Induction of T4 DNA ligase in a recombinant strain of Escherichia coli. Biotechnology and Bioengineering.

[B14] Akesson M, Hagander P, Axelsson JP (2001). Avoiding acetate accumulation in Escherichia coli cultures using feedback control of glucose feeding. Biotechnology and Bioengineering.

[B15] Larsen JEL, Gerdes K, Light J, Molin S (1984). Low copy number plasmid cloning vectors amplifiable by derepression of an inserted foreign promoter. Gene.

[B16] diPasquantonio VM, Betenbaugh MJ, Dhurjati P (1987). Improvement of Product Yields by Temperature-Shifting of Escherichia coli Cultures Containing Plasmid pOU140. Biotechnology and Bioengineering.

[B17] Betenbaugh MJ, Dhurjati P (1990). A comparison of mathematical model predictions to experimental measurements for growth and recombinant protein production in induced cultures of Escherichia coli. Biotechnology and Bioengineering.

[B18] Ryan W, Collier P, Loredo L, Pope J, Sachdev R (1996). Growth Kinetics of Escherichia coli and Expression of a Recombinant Protein and Its Isoforms under Heat Shock Conditions.. Biotechnology Progress.

[B19] Corchero JL, Vila P, Cubarsi R, Villaverde A (1994). Production of thermally induced recombinant proteins relative to cell biomass is influenced by cell density in Escherichia coli batch cultures. Biotechnology Letters.

[B20] Sambrook J, Russel DW (2000). Molecular Cloning (third ed.).

[B21] Bélanger L, Figueira MM, Bourque D, Morel L, Béland M, Laramée L, Groleau D, Míguez CB (2004). Production of heterologous protein by Methylobacterium extorquens in high cell density fermentation.. FEMS Microbiology Letters.

